# Effect of *Millettia ferruginea* (Birbra) foliage supplementation on feed intake, digestibility, body weight change and carcass characterstics of Washera sheep fed natural pasture grass hay basal diet

**DOI:** 10.1186/2193-1801-3-50

**Published:** 2014-01-24

**Authors:** Berhanu Alemu, Getachew Animut, Adugna Tolera

**Affiliations:** Department of Animal Science, Debre Markos University, P.O. Box 269, Debre Markos, Ethiopia; School of Animal and Range Sciences, Haramaya University, P. O. Box 138, Dire Dawa, Ethiopia; School of Animal and Range Science, Hawassa University, P. O. Box 5, Hawassa, Ethiopia

**Keywords:** Body weight, Digestibility, Dry matter intake, Feeding trial, *Millettia ferruginea*, Natural pasture grass hay, Washera sheep

## Abstract

Twenty-four yearling male local Washera lambs with an average initial body weight of 18.14 ± 1.07 kg were used to assess the nutritional value of Millettia ferruginea. Experimental animals were grouped into six blocks of four animals, and each animal was randomly assigned to one of the four dietary treatment feeds. The treatments used were; Sole natural pasture grass hay (T1), and 150, 300, 450 g DM Millettia ferruginea leaf hay with ad libitum natural pasture grass hay assigned for (T2), (T3) and (T4), respectively. The feeding trial was carried out for 80 days followed by a 10 days of digestibility trial. Carcasses of each experimental animal were evaluated at the end of the digestibility experiment. Millettia ferruginea leaf hay had 224.6, 556.6, 360.7and 127.4 g/kg crude protein (CP), neutral detergent fiber (NDF), acid detergent fiber (ADF) and acid detergent lignin (ADL), respectively. The average intakes of Millettia ferruginea leaf hay were 0, 133, 263 and 253 g/day for T1, T2, T3 and T4, in that order. The proportions of Millettia ferruginea leaf hay intake from the total dry matter (DM) were 0, 23.5, 44.1, and 43.3% for T1, T2, T3 and T4, respectively. The total DM intake was not significant but showed a trend of T1 > T3 > T4 > T2. CP intake was higher for T3 and T4 with the least intake for T1. Final body weight measurement was higher for T3 and T1 but lower and negative for T2 and T4. Generally, body weight measurements were not consistent in the supplemented groups throughout the trial period. The weight of heart, spleen, and liver were higher for the supplemented groups compared to the sole grass hay. From the results of the current study, it can be concluded that, Millettia ferruginea had some limiting factors, which prevented the animal from efficiently utilize it. Therefore, this study revealed the indispensable role of animal feeding experiments with target animals to examine such impacts.

## Introduction

Inadequate and fluctuations in feed supply, both in terms of quantity and quality, is the major stumbling-block affecting livestock production in Ethiopia (Legesse 2008). In the mixed crop-livestock production systems of the Ethiopian highlands, feed resources for livestock mainly come from marginal pasturelands, crop residues, and aftermath grazing (Bogale *et al*. 2008; Tsegaye *et al*. 2008). Forages from marginal pasturelands and crop residues are of generally low quality resulting to insufficient nutrient supply, low productivity and even body weight loss of animals when fed alone (Hindrichsen *et al*. 2004). Thus, there is a great need to explore alternate feed resources that could not compete with human food and boost the feeding values of low quality roughages. In this regard, fodder tree leaves are rich in protein, soluble carbohydrates, minerals and vitamins (Bakshi and Wadhwa 2007). The use of tree leaves in ruminant feeds has been reported to enhance microbial growth and rate of degradation and passage rate of digesta (Bonsi *et al*. 1994, 1995). Supplementation of animals on grass basal diets with tree leaves increased feed intake and growth rate of West African dwarf sheep and small East African goats (Aschfalk *et al*. 2002; Rubanza *et al*. 2007). The use of *Leucaena leucocephala* leaf as a supplement has been reported to increase milk production of grazing dairy cattle (Kakengi 2001). Supplementation with forage legumes, which may include herbaceous and shrubby or tree legumes can enhance the utilization of poor quality roughages in smallholder mixed farming systems (Tolera 2007).

The range of crude protein (CP) content of 140 to 290 g/kg dry matter (DM) has been noted for most browse species (Leng 1992), which is above the minimum level of 60 to 80 g/kg DM CP required for efficient rumen fermentation (Van Soest 1994). Although fodder trees have such important nutritional merits, there are also reports that most tropical browse plants has been found to contain some secondary compounds having anti-nutritional property that may limit their wider utilization and nutritional potential (Melaku 2001; Aganga and Tshwenyane 2003; Assefa 2007). For instance, high level of tannin in forages reduced digestibility of CP (Silanikove *et al*. 2001) and lower carcass yield and quality (Priolo *et al*. 2005).

Production of herbaceous and tree forage legumes through integration with food or cash crops to serve as supplemental feeds can be among the potential options to improve nutrient supply to livestock (Alemayehu 2006). In this regard*, Millettia ferruginea* (Hochst.) Baker, locally called ‘Birbra’ is a potential agroforestry multipurpose legume tree species endemic to Ethiopia (Bekele 2007). It is a fast growing species with high coppicing abilities after pruning. It is used as a shade tree for coffee growing regions and used in agroforestry to improve soil fertility (Banouzi *et al*. 2008). It is also used as bee forage (Bekele 2007). As to the survey reports of Negash (1995), the flowers along with leaves are highly valued for fattening goats and sheep in the southern parts of the country. Mekoya (2008) reported that *Millettia ferruginea* leaf contains 911, 260, 623, 433 and 215 g/kg DM, organic matter (OM), crude protein (CP), neutral detergent fiber (NDF), acid detergent fiber (ADF) and acid detergent lignin (ADL), respectively. ILRI (2008) also reported that samples of matured leaves of *Millettia spp.* contain 894.1, 219.0, 405.3, and 227.4 g/kg DM, OM, CP, ADF, and ADL, respectively. Although documented knowledge was not available, farmers who used *Millettia ferruginea* reported that the tree is utilized for growth and fattening purposes of different classes of animals (Alemu *et al*. 2013). Surprisingly, no research work has so far been done on *Millettia ferruginea* in feeding systems for ruminants. Therefore, the objectives of this study were to evaluate the effect of *Millettia ferruginea* leaves on feed intake, digestibility, daily weight gains and carcass characteristics on Washera sheep fed a basal diet of natural pasture grass hay.

## Materials and methods

### Description of the experimental site

The experiment was conducted in Finote Selam town, which is located in northwest Ethiopia, 387 km from the capital Addis Ababa on the main road to Bahir Bar, the capital of Amhara National Regional State (ANRS). The area lies at an altitude of 1800 meters above sea level, and at a latitude and longitude of 10° 41' N and 37° 16' E, respectively. The main rainy season extends from June to September, and the mean annual rainfall of the area is 1250 mm. The average minimum and maximum temperatures are 14°C and 26°C, respectively. The area is characterized by having alluvial, red, and black soils (JTDARDO 2010).

### Experimental feeds preparation and feeding

The experimental feed, *Millettia ferruginea* leaf hay, was collected from Zeghe peninsula, a village found around Bahir Dar town, the capital of ANRS on the other side of Lake Tana. The leaves of *Millettia ferruginea* were collected from trees used as coffee shade by the farmers around mid October 2011. The grass hay was chopped manually to a size approximately 3–6 cm long before providing to the animals. The daily *Millettia ferruginea* foliage supplements was offered in two equal portions at 0800 am and 1600 pm. Grass hay was provided to all animals ad libitum as a basal diet. Individual feed troughs for natural pasture grass hay and *Millettia ferruginea* leaf hay separately, and water troughs were provided for each experimental animal. Animals were accustomed to the experimental diets for 15 days before the commencement of the actual experiment. The actual experiment, the feeding and digestibility trial, took a total of 90 days with 80 days of feeding trial followed by 10 days of digestibility trial.

### Experimental animals and management

Twenty-four intact male yearling Washera sheep with similar body condition and initial body weight of 18.14 ± 1.07 kg (mean ± SD) were bought from a local market of Genet Abo town. Age of the animals was determined by dentition and information obtained by the owners. Animals were neck tagged for identification, and were quarantined for 21 days. During this time, animals were dewormed for internal parasites using albendazol and fasinex and sprayed for external parasites using diazinon. Experimental animals were also vaccinated against pasteurollosis and anthrax based on recommendation of a veterinarian. After the quarantine period, animals were housed individually in separate pens.

### Experimental design and treatments

There were four treatments in this experiment. Treatments were supplementation of *Millettia ferruginea* foliage hay to each sheep at levels of 0 (T1), 150 (T2), 300 (T3) and 450 g DM/day (T4) to experimental sheep fed natural pasture grass hay basal diet. The natural pasture grass hay basal diet consisted of mainly a mixture of *Cynodon* and *Digitaria* species. The experimental design was a randomized complete block design (RCBD). The experimental animals were grouped into six blocks of four animals each based on their initial body weight (IBW) that was determined by taking the averages of two consecutive weights after overnight fasting at the end of the quarantine period. Animals in a block were then randomly assigned to one of the four experimental treatments making six animals per treatment. The daily *Millettia ferruginea* foliage supplement was offered in two equal halves at 0800 and 1600 hours. Natural pasture grass hay was provided to all animals *ad libitum* by adding a 20% allowance of the previous day’s intake*.* All sheep had free access to water and mineralized salt block.

### Chemical composition of treatment feeds

The chemical composition of the feeds used in this study is shown in Table 1. The grass hay used as a basal diet in this study had higher content of NDF and ADF and lower CP, ADL and ash content than that of the *Millettia ferruginea* leaf. The level of CP in *Millettia ferruginea* leaf is quite high justifying its possible feeding value as a protein supplement to feeds containing low level of nitrogen.

**Table 1:** **Chemical composition of feeds offered during the experiment**

Chemical fractions	Composition g/kg feed for DM and g/kg DM for others	
	Natural pasture grass hay	***M. ferruginea*** leaf hay
DM	920.2	925.3
OM	934.3	894.5
CP	70.1	224.6
NDF	717.8	556.6
ADF	409.4	360.7
ADL	99.7	127.4

DM = dry matter; OM = organic matter; CP = crude protein; NDF = neutral detergent fiber; ADF = acid detergent fiber; ADL = acid detergent lignin.

### Feeding trial

The feeding trial was conducted for 80 days. The amount of feed offered and refused was weighed and recorded for each sheep daily. DM and nutrient intakes were determined by difference. Representative samples of feeds offered were collected per batch. Refusal samples for each animal were collected and pooled per treatment. Sub-samples of the feed offered and refusals were used for chemical analysis. The animals were weighed initially and every 10 days afterwards. Body weight (BW) was taken before the morning meal and after overnight fasting of the animals. The average daily body weight gain (ADG) was calculated by dividing differences of the final BW and IBW by the number of feeding days.

### Digestibility trial

The digestibility trial was conducted after the feeding trail with the same animals of the feeding trial. All animals were harnessed with fecal collection bags for the determination of digestibility. The digestibility trail took a total of 10 days with three days of adaptation of carrying the fecal bags. After three days of adaptation, daily total fecal output along with the daily feed offered and refusal were weighed and recorded for seven consecutive days for each animal. Out of the daily total fecal output, 20% was sub-sampled to form a weekly fecal composite sample for each animal and stored at -20°C. Fecal samples were then thawed, thoroughly mixed, sub-sampled, dried at 60°C for 72 hours and ground to pass 1 mm sieve screen and stored pending chemical analysis. Grabs of feed samples from each feed and refusals from each animal were collected each day to make a weekly composite feed sample for each feed and refusal for each animal. The refusal samples were then pooled per treatment. The apparent digestibility coefficient of DM and nutrients were calculated using the following formula.

### Rumen fluid collection

At the end of the digestibility trial, rumen fluid was collected from each animal just before morning meal and at 4 and 8 hours after the morning meal using stomach tube for the determination of ruminal pH and ammonia-nitrogen (NH_3_-N). About 30–40 ml of rumen fluid was collected from each animal using stomach tube. The pH of the rumen fluid was determined for each animal using pocket pH meter immediately after collection. The rumen fluid was then strained using double cheesecloth and transferred into a vial. Three to five drops of concentrated sulfuric acid was added and stored pending chemical analysis. Ammonia nitrogen was determined following the procedures described by FAO (1989).

### Carcass parameters

At the end of the digestibility trial, all experimental animals were slaughtered after an overnight fasting. Slaughter weight was recorded and animals were killed by severing the jugular vein using a knife. Blood was collected and weighed. The esophagus was tied to prevent the back flow of rumen content while suspending the animals for skinning. The skin was flayed carefully to avoid adherence of fat and muscle tissue to the skin, and was weighed along with legs below the fetlock joints. The weights of the kidney and omental fat, lungs, trachea, esophagus, heart, kidneys, liver with gall bladder, spleen, testis and penis, the entire alimentary canal (esophagus, reticulo-rumen, omasum and abomasum, small intestine and large intestine), full gut, empty gut, tail, tongue and head were recorded separately. Empty body weight was determined by subtracting the gut fill from slaughter body weight. Hot carcass weight was determined by excluding contents of the thoracic, abdominal and pelvic cavities, head, skin, feet and tail of the animal. Dressing percentage was calculated on the bases of slaughter weight and empty body weight as ratio of hot carcass weight to slaughter weight and empty body weight, respectively multiplied by 100.

The rib-eye muscle area of each animal was determined by cutting perpendicular to the backbone between 11th and 12th ribs and tracing the left and right rib-eye muscles on plastic paper Galal et al. (1979). The area was measured using mechanical polar planimeter (model series 20). The mean of the right and left cross sectional areas were taken as a rib-eye muscle area measurement. Edible offal (EO) component was taken as the sum of blood, liver, reticulo-rumen, omasum, abomasum, large and small intestine, kidneys and kidney fat, omental fat, heart, tongue and tail. Non-edible offal component (NEO) was computed as the sum of spleen, pancreas, head without tongue, skin and feet, testis and penis, lung, trachea, esophagus and gut content. Usable product (UP) was taken as the sum of hot carcass weight, EO and skin.

### Chemical analysis and statistical analysis

Samples of feeds, refusals, and faeces were dried in a forced draft oven at 60°C for 72 hours, ground to pass through one mm screen and used for chemical analysis. The DM, ash and CP contents were determined according to the procedures described by AOAC (1990). Organic matter (OM) content was determined as 100 - ash. Neutral detergent fiber (NDF), acid detergent fiber (ADF) and acid detergent lignin (ADL) were determined following the procedure of Van Soest and Robertson (1985). Data were analyzed using analysis of variance following the General Linear Model (GLM) procedure of SAS (SAS 2002), with the model consisting of treatment and block. Difference among treatment means were separated using least significance difference (LSD) test, when treatment effect was significant (P < 0.05).

### Ethical approval

This experiment was part of a PhD dissertation of Berhanu Alemu, which was checked and approved by the academic commission of Haramaya University following the universities guideline before the commencement of the experiment.

## Results

### Dry matter and nutrient intake

The hay DM intake of the non-supplemented sheep was greater (P < 0.05) than those supplemented with *Millettia ferruginea* leaf, while values among the supplemented treatments were similar (Table 2). Intake of *Millettia ferruginea* leaf DM was lower for T2 than T3 and T4 (P < 0.05), while values for the latter two treatments were similar (P > 0.05). Intakes of total DM, OM, NDF and ADF were similar (P > 0.05) among treatments. Intake of CP followed a similar trend like that of *Millettia ferruginea* leaf DM and was in the order of T1 < T2 < T3 = T4 (P < 0.05).

**Table 2:** **Daily feed and nutrient intake of Washera sheep fed natural pasture grass hay supplemented with** ***Millettia ferruginea*** **leaf hay**

Intake (g/day)	Treatments				SEM
	T1	T2	T3	T4	
DM					
Grass hay	591^a^	434^b^	336^b^	357^b^	36.47
MFLH	-	133^b^	263^a^	253^a^	19.58
Total	591	567	596	612	34.44
OM	561	525	547	562	32.13
CP	42^c^	65^b^	86^a^	85^a^	2.71
NDF	434	385	386	398	24.61
ADF	257	233	234	244	14.63

^a,b,c^Means within a row with different superscripts differ significantly (P < 0.05); SEM = standard error of the mean; DM = dry matter; OM = organic matter; NDF = neutral detergent fiber; ADF = acid detergent; CP = crude protein; MFLH = *Millettia ferruginea* leaf hay; T1 = sole natural pasture grass hay fed *ad libitum*; T2 = 150 g MFLH + natural pasture grass hay fed *ad libitum;* T3 = 300 g MFLH + natural pasture grass hay fed *ad libitum;* T4 = 450 g MFLH + natural pasture grass hay fed *ad libitum*.

### Apparent dry matter and nutrient digestibility

Apparent DM digestibility was greater (P < 0.05) for T2 than other treatments, while values for T1, T3 and T4 were similar (P > 0.05) among each other (Table 3). Digestibility of OM followed almost a similar trend like that of DM digestibility, but the value for T3 was similar with T2 (P > 0.05). Digestibility of CP was lower (P < 0.05) for T1, while values among the supplemented treatments were similar (P > 0.05). The NDF digestibility was greater for T2 (P < 0.05) than T3 and T4, whereas the value for T1 was similar with other treatments. Digestibility of ADF was lower (P < 0.05) for T3 and T4 than the other two treatments, and values between T1 and T2 and between T3 and T4 was similar.

**Table 3:** **Apparent nutrient digestibility coefficients of Washera sheep fed natural pasture grass hay supplemented with** ***Millettia ferruginea*** **leaf hay**

Nutrients	Treatments				SEM
	T1	T2	T3	T4	
DM	0.53^b^	0.60^a^	0.50^b^	0.46^b^	0.02
OM	0.68^b^	0.73^a^	0.68^ab^	0.64^b^	0.01
CP	0.49^b^	0.69^a^	0.66^a^	0.67^a^	0.04
NDF	0.55^ab^	0.60^a^	0.51^b^	0.46^b^	0.02
ADF	0.50^a^	0.55^a^	0.42^b^	0.38^b^	0.02

^a,b^Means within a row with different superscripts differ significantly (P < 0.05); SEM = standard error of the mean; DM = dry matter; OM = organic matter; NDF = neutral detergent fiber; ADF = acid detergent; CP = crude protein; MFLH = *Millettia ferruginea* leaf hay; T1 = sole natural pasture grass hay fed *ad libitum*; T2 = 150 g MFLH + natural pasture grass hay fed *ad libitum;* T3 = 300 g MFLH + natural pasture grass hay fed *ad libitum;* T4 = 450 g MFLH + natural pasture grass hay fed *ad libitum.*

### Rumen parameters

Ruminal pH right before the morning meal was a neutral pH and was similar among treatments (Table 4). The pH of the rumen was slightly reduced 4 to 8 hours after feeding. At 4 hours after feeding, rumen pH was greater (P < 0.05) for T4 than other treatments. At 8 hours after feeding rumen pH was lower (P < 0.05) for T1 than T3, whereas other means were similar among each other. The concentration of ruminal NH_3_-N was lower (P < 0.05) for T1 than T3 but similar (P > 0.05) for T1, T2 and T4, and T2, T3 and T4 at the 4 hour. The values of ruminal NH_3_-N concentration right before feeding and at 8 hour were similar.

**Table 4:** **Rumen pH and ammonia-nitrogen concentration of Washera sheep fed natural pasture grass hay supplemented with** ***Millettia ferruginea*** **leaf hay**

Rumen parameters	Sample collection hour*	Treatments				SEM
		T1	T2	T3	T4	
Rumen pH	0	7.1	7.1	7.1	7.0	0.07
	4	6.8^b^	6.8^b^	6.8^b^	6.9^a^	0.04
	8	6.7^b^	6.8^ab^	6.9^a^	6.8^ab^	0.06
NH_3_-N (mg/L)	0	16.5	17.3	21.1	17.0	2.94
	4	13.4^b^	26.0^ab^	29.2^a^	26.4^ab^	3.45
	8	11.2	17.1	22.2	19.5	4.12

^a,b^Means within a row with different superscripts differ significantly (P < 0.05); SEM = standard error of the mean; *sample collection hours = Right before the morning meal or 0 hour (0), 4 hours (4) and 8 hours (8) after the morning meal; MFLH = *Millettia ferruginea* leaf hay; T1 = sole natural pasture grass hay fed *ad libitum*; T2 = 150 g MFLH + natural pasture grass hay fed *ad libitum;* T3 = 300 g MFLH + natural pasture grass hay fed *ad libitum;* T4 = 450 g MFLH + natural pasture grass hay fed *ad libitum.*

### Body weight change

Since the animals were blocked based on their initial body weight (BW) before assigning to treatments, initial BW was similar among treatments as expected (Table 5). Final BW and BW change for T1 and T3 was greater (P < 0.05) than for T2 and T4, while values between T1 and T3 was similar and so to between T2 and T4 (P > 0.05). Mean daily body weight gains of experimental animals were almost nil and fail to differ among treatments (P > 0.05).

**Table 5:** **Body weight change and average daily body weight gain (ADG) of Washera sheep fed natural pasture grass hay supplemented with** ***Millettia ferruginea*** **leaf hay**

Parameter	Treatments				SEM
	T1	T2	T3	T4	
Initial BW (kg)	18.49	18.28	18.65	18.12	0.34
Final BW (kg)	19.01^a^	17.86^b^	19.21^a^	17.89^b^	0.40
BW change (kg)	0.52^a^	-0.42^b^	0.56^a^	-0.24^b^	0.27
ADG (g/day)	12.00	-8.00	8.00	-6.00	0.003

^a,b^Means within a row with different superscripts differ significantly (P < 0.05); SEM = standard error of the mean; BW = body weight; ADG = average daily gain; MFLH = *Millettia ferruginea* leaf hay; T1 = sole natural pasture grass hay fed *ad libitum*; T2 = 150 g MFLH + natural pasture grass hay fed *ad libitum;* T3 = 300 g MFLH + natural pasture grass hay fed *ad libitum;* T4 = 450 g MFLH + natural pasture grass hay fed *ad libitum*.

**Figure 1:**
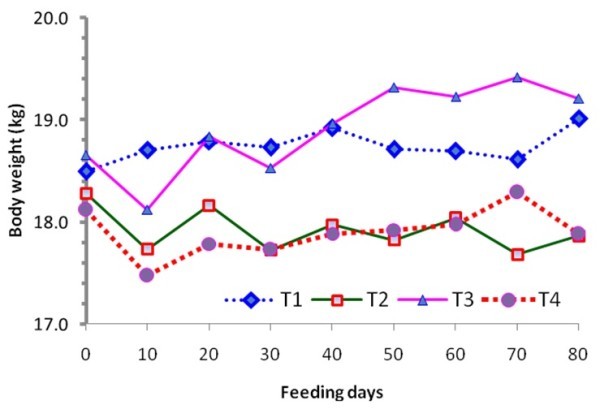
**Trends in body weight change of Washera sheep fed natural pasture grass hay supplemented with different levels of** ***Millettia ferruginea*** **leaf hay.** MFLH = *Millettia ferruginea* leaf hay; T1 = sole natural pasture grass hay fed *ad libitum*; T2 = 150 g MFLH + natural pasture grass hay fed *ad libitum;* T3 = 300 g MFLH + natural pasture grass hay fed *ad libitum;* T4 = 450 g MFLH + natural pasture grass hay fed *ad libitum.*

Trends in body weight change of sheep during the feeding trial are shown in Figure 1. Comparatively, there have been little fluctuations in BW of sheep in the non-supplemented group throughout the feeding trial. Conversely, BW of the supplemented groups tend to highly fluctuate during the study period with little apparent trend.

### Carcass characteristics

Slaughter weight (SW), empty body weight (EBW) and hot carcass weight (HCW) was not affected by treatment (Table 6). Similarly, weights of total edible and non-edible offal and total useable products were similar (P > 0.05) among treatments. Dressing percentage on SW basis was greater (P < 0.05) for T4 than T1 and T2, and on EBW basis was higher for T4 than T1, while other values were similar among each other. Rib-eye muscle area was not significantly impacted by treatment (P > 0.05).

**Table 6:** **Carcass characteristics of Washera sheep fed natural pasture grass hay supplemented with** ***Millettia ferruginea*** **leaf hay**

Parameters	Treatments				
	T1	T2	T3	T4	SEM
Slaughter weight (kg)	18.3	17.6	18.2	17.7	0.82
Empty body weight (kg)	12.2	11.9	12.3	12.3	0.51
Hot carcass weight (kg)	5.8	5.7	5.9	6.0	0.24
Dressing percentage					
Empty body weight basis	47.4^b^	47.8^b^	48.0^ab^	48.9^a^	1.23
Slaughter body weight basis	31.7^b^	32.4^ab^	32.4^ab^	33.9^a^	0.83
Total edible offal (kg)	2.6	2.4	2.6	2.7	0.15
Total non edible offal (kg)	9.9	9.5	9.7	9.0	0.61
Total useable product weight (kg)	10.8	10.5	10.7	10.8	0.45
Rib eye area (cm^2^)	7.3	7.9	7.3	6.6	0.64

^a,b^Means within a row with different superscripts differ significantly (P < 0.05); SEM = standard error of the mean; MFLH = *Millettia ferruginea* leaf hay; T1 = sole natural pasture grass hay fed *ad libitum*; T2 = 150 g MFLH + natural pasture grass hay fed *ad libitum;* T3 = 300 g MFLH + natural pasture grass hay fed *ad libitum;* T4 = 450 g MFLH + natural pasture grass hay fed *ad libitum*.

Out of the edible offal components, only weights of liver, blood, heart, kidney, small intestine, and kidney fat was significantly affected (P < 0.05) by treatment (Table 7). Liver weight was lower in the non-supplemented than the supplemented sheep. Heart weight was greater for T1 than T3, kidney weight was higher for T4 than T1 and T2, weight of small intestine was greater for T4 than T1, and kidney fat was higher for T4 than T2 and T3, while other means were similar among each other. Weight of spleen, esophagus, and penis were the only significantly affected parameter from the non-edible offal components. Spleen was greater (P < 0.05) for T3 than T1, penis was greater (P < 0.05) for T3 than T4 and esophagus was higher (P < 0.05) for T2 than T4 and T1.

**Table 7:** **Edible and non-edible offal components of Washera sheep fed natural pasture grass hay supplemented with** ***Millettia ferruginea*** **leaf hay**

Parameter weight (g)	Treatments				SEM
	T1	T2	T3	T4	
**Edible offals**					
Liver	190^b^	230^a^	250^a^	250^a^	10.87
Blood	712^a^	564^b^	619^ab^	662^ab^	46.65
Tongue	78	76.6	86.4	79.8	7.53
Heart	94.3^a^	84^ab^	80.3^b^	92.0^ab^	5.80
Kidney	51^b^	49^b^	59^ab^	61.3^a^	3.08
Reticulo-rumen	434	403	425	388	28.77
Omasum	62	65	64.1	56.5	4.67
Abomasum	77.0	84.0	90.0	88.3	10.14
Small intestine	276^b^	328^ab^	328^ab^	374^a^	29.01
Large intestine	334.4	304.0	361.5	357.7	45.27
Tail	286	208	230	241	70.38
Kidney fat	19.7^ab^	14.0^b^	13.8^b^	21.0^a^	2.05
Omental fat	10.9	10.8	23.4	16.7	6.59
**Non-edible offals**					
Lung	177	173	184	190	11.34
Skin and feet	2370	2340	2000	2150	223.29
Spleen	24.1^b^	26.7^ab^	28.3^a^	24.7^ab^	1.88
Testicle	125.0	94.0	110.0	82.0	21.51
Penis	28.4^ab^	34.1^ab^	35.9^a^	25.0^b^	3.45
Oesophagus	25.8^b^	38.3^a^	33.8^ab^	23.0^b^	4.75
Trachea	30.9	37.0	27.1	28.3	7.97
Head	1026	1062	1336	1062	159.43
Gut content	6067	5674	5914	5427	405.13

^a,b^Means within a row with different superscripts differ significantly (P < 0.05); SEM = standard error of the mean; MFLH = *Millettia ferruginea* leaf hay; T1 = sole natural pasture grass hay fed *ad libitum*; T2 = 150 g MFLH + natural pasture grass hay fed *ad libitum;* T3 = 300 g MFLH + natural pasture grass hay fed *ad libitum;* T4 = 450 g MFLH + natural pasture grass hay fed *ad libitum.*

## Discussion

### Chemical composition of treatment feeds

The dominant species in the composition of the natural pasture grass hay used as a basal diet in this study were *Cynodon* and *Digitaria*. The fiber fractions, NDF and ADF of these grass mixtures were higher as it was harvested late in the blooming stage compared to the values reported by Skerman and Riverson (1990) and Bediye *et al*. (2007) however, the CP content was almost similar with the values reported by the same authors. Most natural pasture in the highlands of Ethiopia has a CP content below the level (6 – 8%) required for optimum microbial activity (Assefa 2007). However, the average CP content of the natural pasture grass hay used in this study (7.01%) was in the above-mentioned ranges required to support the optimum activity of microorganisms in the rumen (Whiteman 1980; Van Soest 1994; Coleman and Moore 2003). On the other hand, the high CP and relatively low fiber contents of *Millettia ferruginea* leaf hay compared to natural pasture grass hay indicated the potential of this tree to be used as a supplement diet for poor quality roughage in ruminant diets. The *Millettia ferruginea* leaf hay used for this experiment was harvested around the mid of October when the plant was at its full leaf biomass and maturity stage. This stage is found between leaf emergence and leaf shedding periods as the plant is deciduous. At this stage, leaves of *Millettia ferruginea* were well grown, matured and the fiber fractions were relatively higher compared with the stage near leaf emergence (Alemu *et al*. 2013). The average CP content of *Millettia ferruginea* leaf hay (224.6 g/kg DM) in this study was lower than the values (260 g/kg DM) reported by Mekoya (2008) but it was higher than the values (219 g/kg DM) reported by ILRI (2008). The NDF, ADF and ADL fractions of *Millettia ferruginea* in the current study were relatively lower than the values reported by Mekoya (2008), 623, 433 and 215 g/kg DM, respectively. On the other hand, the values of ADF and ADL fractions 405.3 and 227.4 g/kg DM, respectively reported by ILRI (2008) were lower than the values obtained in the current study, whereas the values of ADL 227.4 g/kg DM reported by ILRI (2008) was higher than the values obtained in the current study.

### Dry matter and nutrient intake

In the current study of the feeding trial, majority of the sheep readily accepted *Millettia ferruginea* leaf hay but some of them did not consume the total amount offered. The amount of *Millettia ferruginea* leaf DM consumed by the sheep under T3 and T4 were similar indicating that sheep did not consume *Millettia ferruginea* leaf hay beyond that amount. The proportion of *Millettia ferruginea* leaf hay from the total DM consumed for the supplemented treatment groups were 23.5, 44.1, and 43.3% for T2, T3 and T4, respectively. During the feeding trial period, noticeable physiological disturbances, health problems, and deaths of animals were not manifested because of feeding of *Millettia ferruginea* leaf hay. In this study, significant increase was not observed in the intake of the natural pasture grass hay as the level of *Millettia ferruginea* diet increased. This might be due to the filling or bulk volume of natural pasture grass hay and the *Millettia ferruginea* forage. Kaitho *et al*. (1998), Tolera and Sundstøl (2000) and Assefa (2007) reported similar observations on the intake of tef straw, maize stover and natural pasture hay supplemented with an increasing level of multipurpose tree leaves and herbaceous legumes to sheep. Eroarome (2002) also observed a decrease in the DM intake of goats on the basal diet *Panicum maximum* as the level of *Leucaena leucocephala* leaf increased, regardless of the form of presentation (fresh or wilted). The basal grass DM intake of T1 groups of the current study were higher compared to the results reported by Gashu *et al*. (2009) using similar breeds of sheep fed sole grass hay. The possible reason for these differences in intake could be from the differences in the species as well as the quality of the grasses used in the two experiments. Generally, El hassan *et al*. (2000) and Melaku (2001) point out the difficulty of recommending multipurpose trees as supplement feed merely based on looking at their chemical composition alone as there may be certain limiting factors which may hinder the nutrients from being available to the animals.

### Nutrient digestibility

In the current study, higher apparent DM digestibility coefficient for T2 could be due to the presence of lower level of *Millettia ferruginea* leaf hay in the ration that could be easily diluted by the high level of the basal diet (grass hay). The same possible reason that could substantiate the previous idea may be the existence of low threshold level of the limiting factors in this treatment groups that could not impose negative effects on the digestibility of the dry matter. The apparently higher CP digestibility coefficient for the supplemented group compared to the sole grass hay treatment could be due to the presence of high CP content in the supplement diet than the sole grass hay treatment. On the other hand, absence of difference in apparent CP digestibility among the supplemented groups could clearly indicate the presence of some limiting factors that could hinder efficient CP degradability even when the amount of supplement increased at the level that can supply more protein. The degradability of the fiber fractions, NDF and ADF showed higher value for T2 compared with the rest of the supplemented groups. In most cases, for all parameters of the digestibility coefficients evaluated, the figures for T2 were higher even if there were no statistically significant differences with some of the treatment groups. This obviously indicated the existence of some limiting factors in the supplement diet with the effect that could be aggravated as the level of the supplement diet increased in the ration. The limiting factors such as condensed tannins (CT) prevented the rumen microorganisms as well as the animals from utilizing the nutrients properly.

The content of condensed tannin in the leaves of *Millettia ferruginea* (3.52%) recorded (Alemu *et al*. 2013) was in the level that seemed to create negative effects on the digestibility of feeds in ruminants. Anti-nutritional factors such as CT inhibit plant protein degradation and decrease sulphur availability in the rumen, which in turn affected the digestibility of total tract nitrogen (Animut *et al*. 2008) and plant cell walls (Aganga and Tshwenyane 2003). Duttan et al. (1999) also observed a depression in the value of CP and OM degradability on goats under a basal diet of rice straw supplemented with *Prosopis cineraria* because of the high contents of tannin in the leaves. It is also possible that these tannins inhibit microbial enzymes in the rumen and decrease the availability of plant proteins for digestion in the intestines (Norton 1994). Barry (1987) indicated that the low-level of condensed tannin (2 - 3%) in ruminant diets had beneficial advantages as it reduces the degradation of useful protein in the rumen by the formation of protein-tannin complexes. Therefore, knowledge of concentration of limiting factors in forage legumes and identifying potential inclusion levels in ruminant diets would be a good strategy of utilizing such resource efficiently by decreasing nitrogen excretion (McMahon *et al*. 1999).

### Rumen parameters

The value of pH in the current study was between the optimum ranges of 6.7 - 7.1 required for cellulolysis activity and it was above the level of pH 5.7 required for microbial protein synthesis (Stewart 1977). In addition, Mould and Orskov (1983) and Mould *et al*. (1983) also reported that the inhibitory effect of rumen pH on cellulolysis occurred when the values drop below 6.1. Therefore, based on these suggestions, the values of rumen pH recorded at the different times for the different levels of the treatment diets of the current study may not be inhibitory to the fiber degradation and microbial protein synthesis.

On the other hand, the value of rumen NH_3_-N concentration in the current study was below the range 50–60 mg/l rumen fluid, which is assumed adequate for maximal microbial protein synthesis (Satter and Slyter 1974). The low level of NH_3_-N in the current study for the supplemented groups could arise from the presence of some limiting factors, which may indirectly affect the rumen functions through decreased protein degradation, and this may in turn interfere or depress fibre digestibility (Leng *et al*. 1993).

### Body weight change

The average daily gain of sheep in the current study was not significant for all treatment groups but the values were positive for T1 and T3 but negative for T2 and T4. Body weight loss of animals in the current study was observed more on the supplemented groups than the sheep under sole natural pasture grass hay. From animals grouped under the three levels of supplemental diets, about 57.1% of them lost body weight (four, three and five animals from T4, T3 and T2, respectively), whereas the rest 41.9% did not lose weight, rather some of the sheep gained even up to 20 g body weight/day. In this regard, some researchers, Norton (1994) and Njidda and Ikhimioya (2010), revealed the existence of recent evidence that some rumen microorganisms are able to metabolize tannins or remain active in a high tannin environment and overcome the detrimental effects. On the other hand, the positive body weight balance observed for the groups of animals under the sole grass hay, in the current study indicated the sufficiency of the grass hay to support at least maintenance requirements. Demo (2013) also reported a non-significant body weight change among the supplemented (*Millettia ferruginea* leaf meal) and the control (natural pasture hay) groups in an experiment conducted using local sheep from Gedeo zone, southern Ethiopia.

Generally, fluctuations were observed in body weight changes throughout the feeding trial period of the current experiment. Assefa (2007) also observed a sharp decline in body weight of sheep fed a wheat bran-noug mixture concentrate substituted with *Chamaecytisus palmensis* in the first six weeks of the feeding trial followed by a slow declining trend of body weight until the end of the experiment. The relatively high CP intake and lower gain of sheep under the supplemented treatments compared to the sole grass hay in the current study could be due to the inefficient utilization of CP in the presence of *Millettia ferruginea* leaf hay. This inefficiency of utilization might be due to the presence of some limiting factors as well as a relatively high content of fiber in the natural pasture grass hay and *Millettia ferruginea* leaf hays. Duttan et al. (1999) also observed a weight loss in goats fed a basal diet of rice straw supplemented with *prosopis cineraria* leaves because of the presence of high tannin and high fiber in the leaves.

In the survey work reports of Negash (1995) and Alemu *et al*. (2013), the interviewees responded that fresh forms of *Millettia ferruginea* leaf to be the best diet for fattening of cattle including small ruminants without negative impacts on animals. However, the current study showed negative results with the two survey reports. These disagreements might arise from the form of presentation of *Millettia ferruginea* leaves to the animals as the form of presentation in the current study was in its dry form (Eroarome 2002). Generally, the weight loses occurred on sheep under the supplement diet and the weight balance for groups under sole grass hay leads us to envisage that *Millettia ferruginea* leaves contain some limiting factors, which could possibly hinder the nutrients from being properly utilized by microorganisms and animals at large.

### Carcass parameters

In the evaluation of carcass traits of sheep and goats factors such as nutrition, genotype, age, sex, season and other related factors that can affect growth and carcass traits should be considered (Devendra and Burns 1983; Hagos and Melaku 2007). In the current study, the values obtained in body weight change for the different treatment groups were not reflected on the carcass parameter on the corresponding treatment groups. The high dressing percentage observed for T4 compared with T3 was due to a relatively high carcass weight and lower empty body weight obtained for T4. With regard to the edible offals, in different parts of the country, different proportions of the internal offals including blood are edible and saleable and fetch extra money that could add value to the carcass. Due to differences in test of the offals and eating habits of the people, edible and saleable portions of the offals in one area of a country may not be acceptable in other (Legesse 2001). Among the internal organs, the weight of liver, kidney, spleen, and small intestine were higher for the supplemented groups than the control ones. Betsha (2005) and Moges (2005) also observed significant effect on liver, heart, and kidneys with concentrate supplementation. The high weight of liver of lambs in the supplement group of the current study could be the results of the high level of anti-nutritional compounds such as condensed tannins and the required detoxification processes, which increased the size of the liver (Assefa 2007). Likewise, Hagos and Melaku (2007) also reported an increase in the weight of liver as the supplement diet increased and they suggested that this phenomenon could happen due to the storage of more reserve substances such as glycogen. Gebreyohannes *et al*. (2003) also reported higher liver weight in Horro sheep fed with sesbania forage compared to those fed maize grain and grass hay.

## Conclusions

The high CP and relatively lower fiber fractions of *Millettia ferruginea* leaf showed its potential for use as a supplement to roughage based diets. However, this was not reflected in intake, digestibility, growth rate and carcass output of sheep in this study, possibly due to the presence of anti-nutritional factors in *Millettia ferruginea* leaf. In conclusion, the actual feeding value of *Millettia ferruginea* leaf appeared to be low, and demands further study to identify problems associated with its feeding value.

## Author’s information

BA is a PhD in animal nutrition and currently teaching in the department of Animal Science, Debre Markos University, Ethiopia.

GA is a PhD and associate professor of animal nutrition and currently teaching in the School of Animal and Range Sciences, Haramaya University, Ethiopia.

AT is a PhD and Professor of animal nutrition and currently teaching in the school of Animal and Range Sciences, Hawassa University, Ethiopia.

## Abbreviations

ADF: Acid detergent fibre
ADG: Average daily gain
ADL: Acid detergent lignin
ANF(s): Anti-nutritional factor (s)
ANRS: Amhara National Regional State
AOAC: Association of Official Analytical Chemists
BW: Body weight
CP: Crude protein
DM: Dry matter
DMI: Dry matter intake
EO: Edible offal
FAO: Food and Agriculture Organization of the United Nations
GLM: General linear model
IBW: Initial body weight
ILRI: International Livestock Research Institute
LSD: Least significant difference
NDF: Neutral detergent fiber
NEO: Non edible offal
OM: Organic matter
SAS: Statistical analysis system
SEM: Standard error of the mean.
